# Genetic Diversity of the Cichlid *Andinoacara latifrons* (Steindachner, 1878) as a Conservation Strategy in Different Colombian Basins

**DOI:** 10.3389/fgene.2020.00815

**Published:** 2020-07-24

**Authors:** Luz Elena De la Ossa-Guerra, Mateus Henrique Santos, Roberto Ferreira Artoni

**Affiliations:** ^1^Laboratório de Genética e Evolução (LabGEv), Programa de Pós-Graduação em Biologia Evolutiva, Departamento de Biologia Estrutural, Molecular e Genética, Universidade Estadual de Ponta Grossa, Ponta Grossa, Brazil; ^2^Postgraduate Program in Evolutionary Genetics and Molecular Biology, Federal University of São Carlos, São Paulo, Brazil

**Keywords:** cichlids, isolation, Cauca-Magdalena basin, distribution, conservation

## Abstract

Cichlids constitute a diverse monophyletic group that have developed adaptive strategies to thrive in diverse environments. *Andinoacara* represents an example of diversification on the South American Andean uplift, providing a key model for understanding the evolution of biogeographic patterns. In this study, we analyzed the species *Andinoacara latifrons* using two mitochondrial markers (*COI*, *cytb*) and one nuclear marker (*RAG1*) in a populational level. Sequences were obtained through tissue collection and from the GenBank database. Populational analysis showed significant structuration among populations, also corroborated with population pairwise F_*st*_ results. Fu’s Fs and Tajima’s D results showed populations that seems to be under populational expansion. We identified 22 haplotypes using *cytb*. The population associations in the Cauca haplotype are related to the Momposina depression and the mixture of the Cauca-Magdalena river basins in the lower Cauca-Magdalena region. We constructed a new phylogenetic tree, which grouped mainly two *A. latifrons* lineages: (1) an upper Magdalena and Catatumbo clade and (2) an upper Cauca and upper Magdalena clade. Thus, *A. latifrons* represents a diverse entity that contributes to our understanding of the evolutionary history of northern South America. Our findings provide insight into devising public policies in determining refuges for the preservation of biodiversity in the lower Cauca and Magdalena regions in Colombia.

## Introduction

The Neotropical region shows exceptional species richness and endemism due to diversifications associated with its unique geological history resulting in a complex topography and climatic history, but is still poorly understood (Bagley et al., 2016). The family Cichlidae, which occur in the region, are the most diverse group within the order Perciformes. The Neotropical region is estimated to have around 450 species and 50 genera of cichlids (Kullander, 2003). It represents a monophyletic clade with an incompletely understood phylogeny (López-Fernández et al., 2010) that includes species from Central America, the Antilles, and South America (Smith et al., 2008). Paleontological evidence suggests that this teleost group did not diversify into its recognizable modern families and genera until the Late Cretaceous or Paleogene (Reis et al., 2016). The north of South America shows one of the highest levels of species richness after Brazil, with the ichthyofauna of the Magdalena River located between the eastern and western cordilleras of the Colombian Andes long recognized as characteristic of this richness (Cala, 1987). Geologic processes involved in the formation of the *trans*-Andean region (it includes Panamá, Atrato-Pacific slope, Magdalena and Maracaibo basins) (Albert et al., 2006) contributed to the richness of this region, providing an important framework for addressing phylogeographic connections associated with the hydrographic history of the region (Rincon-Sandoval et al., 2019). In addition, the Andean uplift generated a biological dynamic that allows us to explain life histories that can be elucidated under evolutionary concepts of speciation, geographic isolation, vicariance, population dynamics, and others. *Andinoacara* represents a key example of these dynamics. The evolutionary history of cichlids could have originated from river-dwelling taxa, since they are the most diverse and geographically widespread and postulated to have maintained ancestral geographic patterns and distributions (Kullander, 1988).

In order to understand the *Andinoacara* species cluster, Musilová et al. (2008), confirmed that the polyphyletic group *Aequidens pulcher rivulatus* represents an unnamed genus with strong support through phylogenetic analysis. Subsequently, Musilová et al. (2009) designated this group as a new genus (*Andinoacara*) belonging to the tribe Cichlasomatini that includes eight valid species (Musilová et al., 2009). The processes of diversification within the *Andinoacara* group could have been governed by vicariance events, as is the case in many cichlids in the trans-Andean region. Thus, the distribution of *Andinoacara* ranges from the Magdalena Basin to the trans-Andean region of South America, including the northeast *trans*-Andean region (Maracaibo basin) and the south of Lima in central Perú (Musilová et al., 2015).

*Andinoacara latifrons* was originally described within *Aequides*, a genus consistently designated as a polyphyletic group (Musilová et al., 2008; Smith et al., 2008). Musilová et al. (2015) reconstructed the history of some trans-Andean cichlids using the mitochondrial gene cytochrome b (*cytb*), including several species in the genus *Andinoacara*. In this study, they found that *A. latifrons* form separate clades in Magdalena, Sinú, and Cauca, supporting allopatry by mtDNA. The synonyms of *Andinoacara latifrons* are *Aequidens latifrons* and *Acara latifrons*, and the type locality of the species is the Magdalena River, Colombia. The distribution of *A*. *latifrons* is centered in Colombia, in the Magdalena, Atrato, Sinú, Cauca, and San Juan basins (Kullander, 1998). The species reaches a maximum length of 17 cm. The conservation status of these species is least concern and there are no conservation measures in the habitat regions for this species. It does not occur in protected areas, but some areas of presence of this species are in risk of thread due to contamination of some industries like mining and petroleum. Mining projects have been developing in the region of the Upper Magdalena which in some years could generate an effect of loss of biodiversity, including the endemic fauna. This reason and the poor studies of the dynamic of this species, including reproduction, behavior, and the use of genetic and molecular markers for populational analysis allows designate to *A. latifrons* as a reference specie to preserve areas as the Depresión Momposina by anthropization damages.

To better understand the taxonomic and biogeographic evolutionary history of the species, it is important to understand and to analyze genetic patterns and the influence of the processes of diversification driven by geographic barriers. This is important insofar endemic species support the diversity of a region by their richness, also, population studies can help to recognize genetic patterns that show the level of mixture and structuration; it can be useful to track the states of endemic species like *A. latifrons* and avoid its extinction in some areas or habitat degradation.

In this study, we analyzed the genetic and haplotype diversity of *A. latifrons* at the population level using mitochondrial [cytochrome oxidase 1 (*COI*) and *cytb*] and nuclear [recombination activating gene (*RAG1*)] markers. As *A. latifrons* is an important model species, evolutionarily significant units (ESUs) (Ryder, 1986) could be established for conservation purposes, as well as to elucidate evolutionary processes that reflect the history of drainage basin formation.

## Materials and Methods

### Sample Collection

The material used and amplified for posterior analysis was acquired from the Ichthyological section of the Zoological Collections of the University of Tolima [Colección Zoológica Universidad del Tolima, Ictiología, Ibagué, Colombia (CZUT-IC)]. We also used *cytb* sequences from the GenBank database (Clark et al., 2016). Detailed information of specimens (including region, authors, accession number, and gene) are summarized in Supplementary Material 1.

### DNA Extraction and Amplification

DNA was extracted from muscle tissue preserved in 96% ethanol through the phenol-chloroform method (Sambrook and Russell, 2006). To perform molecular systematics analysis, the mitochondrial genes *COI* and *cytb*, and the nuclear gene *RAG1* were used. To amplify ca. 610 pb *COI* we used the primers Fish*F*1 5′-TCAACCAACCACAAAGACATTGGCAC-3′ and Fish R1 5′-TAGACTTCTGGGTGGCCAAAGAATCA-3′ (Ward et al., 2005). The PCR conditions in 25 μL were as follows: 2.5 μL of Buffer (10X), 1.25 μL of MgCl_2_ (50 mM), 0.5 μL of mix dNTPs (10 mM), 0.5 μL of each primer (10 mM), 0.2 μL of Polymerase Taq (1U), 17.3 μL of ultrapure water and 2.5 of DNA template (50 ng/μL). The thermal profile consisted of an initial denaturation of 2 min at 94°C, followed by 35 cycles of 30 s at 94°C, 45 s at 52°C and 1 min at 72°C, and a final extension of 10 min at 72°C. The fragment of 940 pb of the gene *cytb* was amplified with primers Fishcytb-F 5′-ACCACCGTTGTTATTCAACTACAAGAAC-3′ and Truccytb-R 5′-CCGACTTCCGGATTACAAGACCG-3′ (Sevilla et al., 2007). The PCR conditions in 25 μL were: 2.5 μL of Buffer (10X), 0.8 μL of MgCl_2_ (50 mM), 0.5 μL of mix dNTPs (10 mM), 0.5 μL of each primer (10 mM), 0.2 μL of Polymerase Taq (1U), 18.1 μL of ultrapure water and 2.5 of DNA template (50 ng/μL). The thermal profile consisted of an initial denaturation of 2 min at 94°C, followed by 36 cycles of 1 min of denaturation at 94°C, 1 min at 55°C, 1 min at 72°C, and a final extension of 8 min at 72°C. The nuclear gene *RAG1* was amplified with the primers forward 5′-CTGAGCTGCAGTCAGTACCATAAGATGT-3′ and reverse 5′-CTGAGTCCTTGTGAGCTTCCATRAAYTT-3′ (Grande et al., 2004). The conditions and the thermal profile to amplify the 1481 pb of PCR product were the same as those used for the *cytb* gene. The PCR products were purified with the Kit Illumina GFX^TM^ PCR DNA and Gel Band Purification (GE HealthCare), following the manufacturer’s instructions. PCR products were sequenced by Macrogen Inc. (Korea) and ACTGene Análises Moleculares (Rio Grande do Sul, Brazil). After sequencing, each electropherogram was verified manually.

### Populational Analysis

We selected the *cytb* marker to perform haplotype and genetic analysis due to its large number of sequences, we use 55 sequences for analysis (see Supplementary Material 1). We performed an analysis of molecular variance (AMOVA) considering seven groups according to proximity in basins [Group 1 (loMg, Nechi, Jorge, loCau); Group 2 (Sinu); Group 3 (Cesar, SNSM); Group 4 (UpCau); Group 5 (UpMg); Group 6 (Atrato); Group 7 (Catatumbo)], population pairwise F_*st*_, and Fu’s Fs and Tajima’s D statistics using the Arlequin software (Excoffier et al., 2007). A haplotype network was constructed using the TCS method (Clement et al., 2000) implemented in the software PopArt v.1.7 (Leigh and Bryant, 2015). Populations were labeled as: loMg, lower Magdalena; Nech, Nechí; Sinu, Sinu; Ces, Cesar; UpCau, Upper Cauca River; Jorge, San Jorge River; UpMg, Upper Magdalena; Atr, Atrato; Cat, Catatumbo River; SNSM, coastal rivers of Sierra Nevada de Santa Marta; loCau, lower Cauca.

### Phylogenetic Analysis

A Bayesian tree was constructed using a concatenated matrix from the genes *COI*, *cytb*, and *RAG1* with a sample of 15 sequences and a total of 2,778 pair bases (see Supplementary Material 1). We ran 500,000 MCMC simulations, discarding a 20% burn-in. The Akaike Information Criterion, corrected (AICc) was used to select the model of molecular evolution (*COI* = HKY and *cytb* = TN93 + 5 gamma categories; *RAG1* = K2 + 5 gamma categories) using the jModelTest software (Posada, 2008). Mr. Bayes v.3.2.6 (Huelsenbeck and Ronquist, 2001) was used to construct the tree. Phylogenetic trees from *cytb*, *COI*, and *RAG1* were calculated and also documented in Supplementary Material 2. Clade visualization and editing was performed in Fig Tree v1.4.2 (Rambaut, 2012). Adobe Photoshop CS6 (v 13.0) was used to improve the trees output from the analyses for use in subsequent figures.

## Results

DNA sequences of 907 pb of the mitochondrial *cytb* gene were used from a total of 55 sequences (16 sequences from this study and 35 sequences from Musilová et al., 2009), a fragment of 436 pb of the mitochondrial gene *COI* (16 sequences), and 1436 pb from a nuclear gene *RAG1.* The nucleotide diversity of the entire dataset of each gene were *cytb* = 0.021, *COI* = 0.20, and *RAG1* = 0.0024.

### Populational Analysis

The statistical results of nucleotide diversity, Fu’s Fs and Tajima’s D indexes for the *cytb* gene are summarized in Table 1. Negative values for the Fu’s Fs index show purifying selection or populational expasion (lower Magdalena, Nechí, Sinu, San Jorge, Upper Magdalena river), while positive values (Ces and Atr) represent deficiency of alleles; non-calculated values in some populations can be related with presence of unique sequences. The Tajima’ D also reinforce the populational expansion in the lower Magdalena, Sinú, and San Jorge populations. The AMOVA showed the highest variation among population and groups (64.49%) (Table 2); also, fixation indexes were more significant among and within populations. Population pairwise Fst results are showed in the Table 3, the highest values between populations are related with the higher distances among rivers in the different basins.

**TABLE 1:** Summarized data of the gene *cytb* for each population.

**Population/Haplotype name**	**Location**	**Sample size**	**Nucleotide diversity**	**Fu’s Fs**	**Tajima’s D**
Lower Magdalena/loMg	Lower Magdalena river	8	0.000741 ± 0.000714	–12.73	–0.44794
Nechí river/Nech	Nechí river	5	0.004263 ± 0.002782	–3.64	1.02876
Sinu river/Sinu	Sinu river	10	0.001528 ± 0.001154	–0.17	–1.83913
Cesar river/Ces	Cesar river	3	0.005822 ± 0.004803	0.45	0
Upper Cauca river/UpCau	La Vieja river, Piedra de Moler	7	0	−	0
San Jorge river/Jorge	San Jorge river	6	0.001674 ± 0.001337	–0.98	–0.67613
Upper Magdalena river/UpMg	Anchique river	5	0.009825 ± 0.006384	–0.47	1.82778
Atrato river/Atr	Humedal Gavilán (Carmen de Apícala)	2	0.005459 ± 0.005980	1.60	0
Catatumbo river/Cat	Quebrada Tabrio	1	0	−	0
Coastal rivers of Sierra Nevada de Santa Marta/SNSM	Cañas river	2	0	−	0
Lower Cauca river/loCau	Lower Cauca river	4	0	0.00000309	0

**Nucleotide diversity and the statistical Fu’s Fs and Tajima’s D are calculated.**

**TABLE 2:** Analysis of molecular variance AMOVA using *cytb* gene.

**Source of variation**	**d. f.**	**Sum of squares**	**Variance components**	**Percentage of variation**	**Fixation index**
Among groups	6	85, 72	0, 239	6, 19	F_CT_: 0.06186
Among populations Among groups	4	56, 58	2, 50	64, 49	F_SC_: 0.68740*
Within populations	44	50, 05	1, 13	29, 3	F_ST_: 0.70673*

*Significance level 0.05.

**TABLE 3:** Population pairwise F_*st*_.

**Pop**	**1**	**2**	**3**	**4**	**5**	**6**	**7**	**8**	**9**	**10**	**11**
loMg		+	+	+	+	+	+	+	+	+	–
Nech	0.74271		–	+	+	+	–	–	–	+	–
Sinu	0.88273	0.22010		+	+	+	+	–	+	–	–
Ces	0.94388	0.54363	0.86217		+	+	–	+	+	+	–
UpCau	0.89683	0.57270	0.69324	0.87404		+	+	+	+	–	–
Jorge	0.80130	0.33066	0.51327	0.73554	0.68967		–	+	–	–	–
UpMg	0.93741	0.61072	0.84120	1.00000	0.86527	0.36842		+	+	-	–
Atr	0.95647	0.28070	0.11050	1.00000	0.79042	0.68539	1.00000		+	+	–
Cat	0.71014	0.38237	0.48146	0.55610	0.60597	0.37907	0.50276	0.56006		–	–
SNSM	0.89085	0.43512	0.63924	0.86159	0.28222	0.43145	0.76190	0.84914	0.36826		–
loCau	0.95587	0.70290	0.88916	1.00000	0.89474	0.65217	1.00000	1.00000	−0.36364	0.67742	

*p = 0.05. Significant values are showed in the upper diagonal with the plus (+) sign.

**FIGURE 1:**
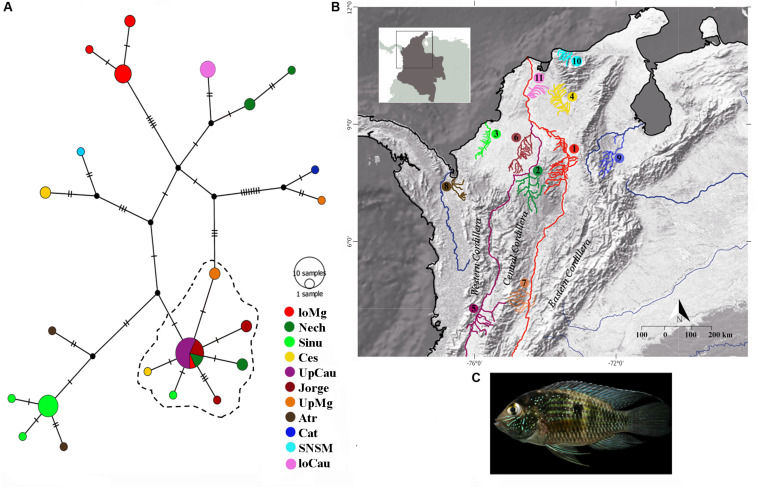
Haplotype distributions of *Andinoacara latifrons* constructed using the mitochondrial gene cytochrome b (*cytb*). **(A)** Haplotype network with main localities; dotted lines indicate most similar haplotypes. **(B)** Geographic distribution of samples. loMag, lower Magdalena; Nech, Nechí; Sinu, Sinu; Ces, Cesar; UpCau, Upper Cauca river; Jorge, San Jorge river; UpMg, Upper Magdalena; Atr, Atrato; Cat, Catatumbo river; SNSM, coastal rivers of Sierra Nevada de Santa Marta; loCau, lower Cauca. **(C)** Photo: *Andinoacara latifrons.*

For the mitochondrial gene *cytb*, we found 84 segregating sites, and 66 parsimony-informative sites; the Tajima’s D of the entire dataset showed a result of D = -0.393, indicating an excess of low frequency of polymorphic sequences, it could signify possible expansion or purifying events, as showed specifically in Table 1 for each population. Likewise, we found 22 different *cytb* haplotypes. Haplotypes results indicate little unique share haplotypes between populations (Figure 1), and slight differences with the UpCauca + San Jorge + Nechí + Cesar + Sinu + UpMg haplotypes constitute an important structure belonging to the Cauca River basin, and could be considered the main ancestor haplotype (the dotted haplotype; Figure 1). It means that the region of the Upper Cauca located on the Western Cordillera and the Upper Magdalena located on the Eastern Cordillera represent an ancient haplogroup with colonizations to the downstream in the past. In addition, direct associations of the populations UpMag and Catatumbo are related to the history of the two basins, due to the uplifting of the central and eastern Cordilleras. The Catatumbo basin produced a more isolated group, induced by a higher rate of mutational steps, mainly explained by distance isolation.

### Phylogenetic Analysis

**FIGURE 2:**
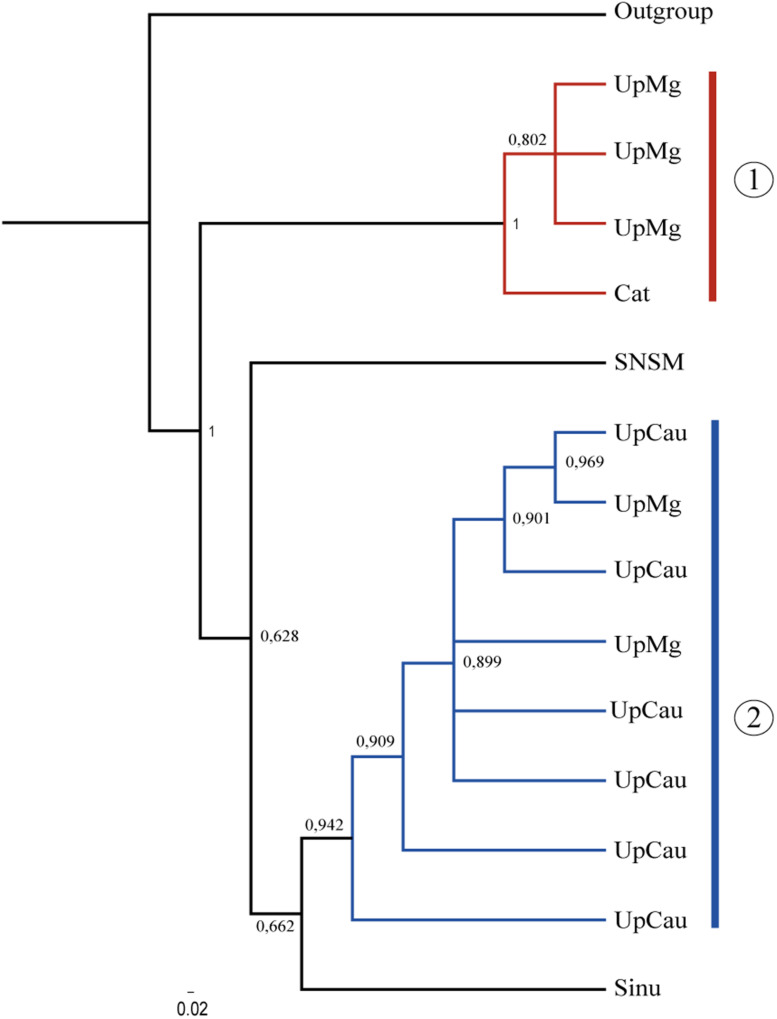
Phylogenetic tree of *A. latifrons* constructed with the mitochondrial genes: *cytb* + COI and the nuclear rag1. Branch support values are represented in Arabic numbers. Sinu, sinu; UpMg, Upper Magdalena; Cat, Catatumbo; SNSM, Coastal rivers of Sierra Nevada de Santa Marta; loCau, lower Cauca; UpCau, Upper Cauca; Outgroup, *Geophagus steindachneri.* The two main clades are shown.

The concatenated tree grouped two main clades with the highest supporting values (Figure 2): (1) a UpMg + Cat clade and (2) a clade from UpCau + UpMg, representing mostly the Magdalena and Cauca river basin, respectively. Separate trees from *cytb*, *COI*, and *RAG1* are in Supplementary Material 2, showing similar topologies among them. Even without a large number of sequences, the combination of mitochondrial (*cytb* + *COI*) and nuclear (*RAG1*) genes highlighted these main clades that correspond to the geological and biogeographic history of the region and shows that the evolution of *A. latifrons* was driven by the geological history of the drainage basin, corroborating the proposal of Musilová et al. (2008). The two main clades described can be lineages seen as evolutionary units which represent important river basins, principally the Magdalena-Cauca river basin that is constantly under contamination and pollution caused by human actions.

## Discussion

The trans-Andean species *A. latifrons*, as type species of the genus *Andinoacara* (Musilová et al., 2009), represents an endemic entity with distribution in the Magdalena, Cauca, and Caribbean basins, as well as the Catatumbo River. The haplotype diversity of the *cytb* gene from the Colombian populations have proven to be congruent with the geological history of this region. Thus, different haplotypes allow us locate each population in a determined region where the higher structuration appears in the farthest places which the genetic flux is lower (e.g., haplotype of the SNSM and Cat) of the ancestral haplotypes of UpMg and UpCau as corroborated with F_*st*_ values (Table 3). This framework can help to inform public policies for the conservation of Colombian biomes through evidences of some species like *A. latifrons*, where the most isolated haplotypes constitute important diversity units of this species.

In this study, the populations of the upper Magdalena-Cauca had an association with the Caribbean basins, but not too strong which is explained by the configuration and presence of *Serranias*, *Sierras*, and mountains, isolated from the Colombian Andes. This association allow us to draw explanations on aspects of the biological evolution of these populations (Castaño-Uribe, 1999). The well supported UpMg + Cat and UpCau + UpMg clades (Figure 2) in the Bayesian tree may explain precisely this configuration around the natural history of the Andes and the dynamics of freshwater fauna highlighting also, an ancient lineage that converge with the original description of *A. latifrons* from Magdalena river basin.

The convergence zone in the *Depresión Momposina* (Herrera et al., 2001) allows some admixture of populations from the lower Cauca-Magdalena basin with populations around this depression (San Jorge, Nechí and lower Magdalena, Figure 1) with populations located more upstream. It constitutes a hybridization area that can validate some of the Andean orogeny and the previous age of populations of *A. latifrons*. However, population structuring is seen in many populations when compared to each other (Table 3). This conclusion was drawn from the comparison of the ages of the last orogenic processes in the Andean Cordillera (12–10 Ma) and the vicariance of *A. latifrons* and *A. pulcher* (∼3.3 Ma).

The Sierra Nevada de Santa Marta (SNSM) is surrounded by the Caribbean Sea to the north, the Cesar River Valley to the southeast, and the Magdalena River to the southwest (Macdonald and Hurley, 1969). This geographic setting helps explain the shared histories of the populations of Ces and SNSM (Figure 1) and also, the structuration of the SNSM and UpMg populations. The continental margin of the Caribbean has been exposed to a wide variety of orogenic processes (Reyes et al., 2004) that have invoked changes in water bodies, consequently influencing the associated fauna. Based on a range of information on organism dynamics, the use of whole-genome sequence (WGS) data offers a high molecular resolution but is costly (Cushman et al., 2018). In order to generate an approximation of evolutionary histories and genetic inferences, we selected to use mitochondrial and nuclear genetic markers that offered a base line and good resolution of haplotype histories in *A. latifrons.* This is useful not only in ecological terms but also to subsequently understand behavioral, reproductive, morphological and phylogenetic traits of different populations. Thus, as a first approach to understand the complexity of this species and its behavior as an endemic species, it was quite efficient to know at least a little, from the genetic and phylogenetic point of view, the distribution and possible expansion events of *A. latifrons*. In addition, it reinforces the idea of the richness in the Colombian basins and how they support the existing biodiversity.

## Implications for Conservation

Developing conservation strategies to establish priority units has historically been difficult. Genetic studies are constantly changing what we know about evolutionary processes. For this reason, the International Union for Conservation of Nature (IUCN, 2020) considers genetic variability an important factor in planning conservation programs (McNeely et al., 1991). It is estimated that the world has more genetic variability than we currently know of, and therefore taxonomic units have to be conserved considering these aspects.

Frankham (2010) highlights three contribution levels made by genetics and molecular biology to conservation genetics. The first level is the conservation objective. Secondly, genetic factors contribute to the risk of extinction through inbreeding depression, loss of genetic diversity, and loss of evolutionary potential. Thirdly, genetic diversity impacts the survival of ecosystems, their function, and their diversity. When we think about these considerations, we need to discuss the units to be conserved, so that ESUs are considered as stocks that deserve separate management and have a high conservation priority. To preserve evolutionary processes, management must preserve natural networks of genetic connections between populations, rather than just as isolated populations within that network (as we point in the Colombian *Depresión Momposina*). Genetic techniques are also essential, because they provide estimates of genetic flow between populations and this could guide efforts to maintain historical levels of genetic exchange between populations (Crandall et al., 2000).

Genomic data confer better results than microsatellite data to delineate Conservation Units (CUs) since genomic data can quantify adaptive variation (Funk et al., 2012). However, more work is needed in the development of new analyses to delineate CUs and to test adaptive differentiation. For example, an ideal analysis could delineate the ESUs and Management Units (MUs), and quantify the adaptive differentiation between these different hierarchical CUs in a single analytical framework, analogous to the current Bayesian MCMC approaches developed to simultaneously infer phylogenies, time of divergence and historical demographic parameters of the given sequence (Drummond et al., 2012). This focus could be important in delineating conservation strategies in the Magdalena-Cauca and Caribbean basins of Colombia mainly by the increasing of mining.

Fish living in high energy and highly oxygenated mountain rivers, such as *Astroblepus* (Astroblepididae), *Chaetostoma* (Loricariidae), and *Trichomycterus* (Trichomycteridae) (Reis et al., 2016), are ecologically important in the upper part of the Magdalena basin. *Andinoacara latifrons* is a species targeted by artisanal fishermen, although the IUCN does not consider it as at risk. However, the IUCN states that the species could be affected by sedimentation and pollution related to deforestation (logging, land use by agriculture, and livestock) and to a lower degree, mining (through associated pollution and sedimentation) that could also affect the drainage of the Magdalena at medium and low altitudes. Just below the Magdalena, in the Momposina basin, ∼80% of the *Ciénagas* are concentrated in depressions in the rocky base of the Late Cretaceous deposits, with stagnant or river-dependent bodies of water accumulating sediments. The muddy plumes of the Magdalena River and the *Canal del Dique* greatly affect the quality of the water, and the combination of mud and reduced salinity may have caused serious damage to coral reefs near where the river flows into the ocean. Data indicate that the Magdalena River contributes ∼10% of the total sediment load discharged from the east coast of South America (Restrepo et al., 2006).

Morphological adaptations of Andean fishes have helped them to colonize and survive at different altitudes (Anderson and Maldonado-Ocampo, 2011). This contributes to the higher richness of Andean fishes found in Colombia. Although cichlids are not the largest teleost group in the region, they are of importance to conservation; in addition, their evolutionary history in South America is correlated to the orogenic history of Andes, this they are considered the ideal model animals for evolutionary study. The high diversity of cichlids and recent environmental degradation in this region suggest that separate conservation strategies are needed for different basins (Figure 1). *Andinoacara latifrons* is found on flood plains and in water that is directly influenced by sedimentation and oxygenation processes. These factors have the greatest impact in the low Magdalena-Cauca region with important repercussions occurring in the Caribbean, including corals reef systems. *Andinoacara latifrons* is an abundant species that highlights the evolutionary history of the Colombian Andes, and that is being affected by environmental contamination. The niche preference of this non-migratory species makes its conservation important and suggests the need for implementation of public policies that promote good practice in mineral extraction.

## Conclusion

In conclusion, our study highlights the importance of recognizing genetic diversity in endemic species, such as *A. latifrons*, not only for the conservation of these species, but also to better understand the taxonomy and historical pattern of geographical colonization. Our findings provide insight into devising public policies in determining refuges for the preservation of biodiversity, including regions of the Lower Cauca and Magdalena river basins in Colombia.

## Data Availability Statement

The datasets generated for this study can be found in the GenBank MN514625, MN514615, MN514616, MN514613, MN514614, MN514612, MN514611, MN514623, MN514619, MN514621, MN514624, MN514609, MN514622, MN514620, MN514618, MN514610, MN514617, MN563598, MN563588, MN563590, MN563585, MN563586, MN563584, MN563583, MN563596, MN563592, MN563594, MN563597, MN563579, MN563595, MN563593, MN563591, MN563582, MN563599, MN514595, MN514592, MN514590, MN514597, MN514594, MN514600, MN514596, MN514599, MN514598, MN514602, MN514606, MN514591, MN514608, MN514601, MN514593, MN514607, and MN514589.

## Ethics Statement

The animal study was reviewed and approved by the COEP (Comissão de Ética em Pesquisa, Sub-Comissão de Ética em Animais) Universidade Estadual de Ponta Grossa.

## Author Contributions

LD performed the analysis under supervision of RA and MS. All authors helped in the writing and analysis of the manuscript.

## Conflict of Interest

The authors declare that the research was conducted in the absence of any commercial or financial relationships that could be construed as a potential conflict of interest.
