# Supplementary material for: Genetic Diversity of the Cichlid Andinoacara latifrons (Steindachner, 1878) as a Conservation Strategy in Different Colombian Basins
Source: Front Genet. 2020 Jul 24;11:815. doi: 10.3389/fgene.2020.00815 (PMC7393254; doi:10.3389/fgene.2020.00815)
Supplement: Supplementary file 1 [file Table_1.DOCX]

Supplementary Material

# Supplementary Table 1

Sequences of the mitochondrial gene Cytochrome b from Genbank database for construction of haplotype network.

| Genbank Code | Voucher | Basin | Author |
| --- | --- | --- | --- |
| KJ127456.1 | loMagd054 | lower Magdalena | Musilová et al. 2015 |
| KJ127455.1 | loMagd051 | Lower Magdalena | Musilová et al. 2015 |
| KJ127453.1 | loMagd055 | Lower Magdalena | Musilová et al. 2015 |
| KJ127449.1 | Nech074 | rio Nechí | Musilová et al. 2015 |
| KJ127448.1 | Nech072 | rio Nechí | Musilová et al. 2015 |
| KJ127447.1 | Nech073 | rio Nechí | Musilová et al. 2015 |
| KJ127445.1 | Jorg041 | Rio San Jorge | Musilová et al. 2015 |
| KJ127442.1 | Sinu013 | Rio Sinú | Musilová et al. 2015 |
| KJ127441.1 | Sinu014 | Rio Sinú | Musilová et al. 2015 |
| KJ127440.1 | Sinu012 | Rio Sinú | Musilová et al. 2015 |
| KJ127439.1 | Jorg031 | Rio San Jorge | Musilová et al. 2015 |
| KJ127436.1 | Jorg032 | Rio San Jorge | Musilová et al. 2015 |
| KJ127434.1 | Jorg034 | Rio San Jorge | Musilová et al. 2015 |
| KJ127388.1 | Cesa08 | rio Cesar | Musilová et al. 2015 |
| KJ127387.1 | Cesa03 | rio Cesar | Musilová et al. 2015 |
| KJ127386.1 | Cesa02 | rio Cesar | Musilová et al. 2015 |
| KJ127385.1 | Jorg13 | Rio San Jorge | Musilová et al. 2015 |
| KJ127384.1 | Jorg12 | Rio San Jorge | Musilová et al. 2015 |
| KJ127378.1 | Sinu09 | rio Sinú | Musilová et al. 2015 |
| KJ127377.1 | Sinu10 | rio Sinú | Musilová et al. 2015 |
| KJ127376.1 | Sinu08 | rio Sinú | Musilová et al. 2015 |
| KJ127375.1 | Sinu07 | rio Sinú | Musilová et al. 2015 |
| KJ127374.1 | Sinu06 | rio Sinú | Musilová et al. 2015 |
| KJ127363.1 | Nech01 | rio Nechí | Musilová et al. 2015 |
| KJ127362.1 | Nech02 | rio Nechí | Musilová et al. 2015 |
| KJ127361.1 | Nech03 | rio Nechí | Musilová et al. 2015 |
| KJ127360.1 | Nech04 | rio Nechí | Musilová et al. 2015 |
| KJ127359.1 | loMagd07 | Lower Magdalena | Musilová et al. 2015 |
| KJ127358.1 | loMagd06 | Lower Magdalena | Musilová et al. 2015 |
| KJ127357.1 | loMagd04 | Lower Magdalena | Musilová et al. 2015 |
| KJ127356.1 | loMagd03 | Lower Magdalena | Musilová et al. 2015 |
| KJ127355.1 | loMagd02 | Lower Magdalena | Musilová et al. 2015 |
| KJ127323.1 | Cauc05 | rio Cauca | Musilová et al. 2015 |
| KJ127322.1 | Cauc04 | rio Cauca | Musilová et al. 2015 |
| KJ127321.1 | Cauc02 | rio Cauca | Musilová et al. 2015 |

# Supplementary Table 2

Sequences obtained from tissue of CZUT-IC (Colección zoológica de la Universidad del Tolima-Ictiología), Colombia.

| Voucher  Catalog number | Tissue number | Basin | Hydrographic region | Accession Genbank Number | | |
| --- | --- | --- | --- | --- | --- | --- |
|  |  |  |  | Cytb | COI | RAG1 |
| None | LGT23 | Quebrada NN-Tabrio | Valle del Cauca, Rio Cauca | MN514625 | MN563598 | MN514595 |
| None | 662 | Río Anchique | Magdalena | MN514615 | MN563588 | MN514592 |
| None | 702 | Río Anchique | Magdalena | MN514616 | MN563590 | MN514590 |
| 15787 | 508 | Carmen de Apicala, Humedal Gavilan | Tolima | MN514613 | MN563585 | MN514597 |
| None | 550 | Río Cañas | Caribe | MN514614 | MN563586 | MN514594 |
| 15787 | 507 | Carmen de Apicala, Humedal Gavilan | Tolima, Upper Magdalena | MN514612 | MN563584 | MN514600 |
| 15787 | 506 | Carmen de Apicala, Humedal Gavilan | Tolima, Upper Magdalena | MN514611 | MN563583 | MN514596 |
| 17045 | L7 | Rio La Vieja, Piedra de Moler | Valle del Cauca, Upper Cauca | MN514623 | MN563596 | MN514599 |
| 17045 | L10 | Rio La Vieja, Piedra de Moler | Valle del Cauca, Upper Cauca | MN514619 | MN563592 | MN514598 |
| 17045 | L12 | Rio La Vieja, Piedra de Moler | Valle del Cauca, Upper Cauca | MN514621 | MN563594 | MN514602 |
| 17045 | L8 | Rio La Vieja, Piedra de Moler | Valle del Cauca Upper Cauca | MN514624 | MN563597 | MN514606 |
| None | 11258 | Desague la española | Magdalena | MN514609 | MN563579 | MN514591 |
| 17045 | L4 | Rio La Vieja, Piedra de Moler | Valle del Cauca, Upper Cauca | MN514622 | MN563595 | MN514608 |
| 17045 | L11 | Rio La Vieja, Piedra de Moler | Valle del Cauca, Upper Cauca | MN514620 | MN563593 | MN514601 |
| 17045 | L1 | Rio La Vieja, Piedra de Moler | Valle del Cauca, Upper Cauca | MN514618 | MN563591 | MN514593 |
| None | 203 | R. Sinu-Isla Blanca-01 | Cordoba | MN514610 | MN563582 | MN514607 |
| None | 755 | Quebrada Batatas | Magdalena | MN514617 | MN563599 | MN514589 |
