# Supplementary material for: Genetic Diversity of the Cichlid Andinoacara latifrons (Steindachner, 1878) as a Conservation Strategy in Different Colombian Basins
Source: Front Genet. 2020 Jul 24;11:815. doi: 10.3389/fgene.2020.00815 (PMC7393254; doi:10.3389/fgene.2020.00815)
Supplement: Supplementary file 2 [file Table_2.DOCX]

Supplementary Material 2


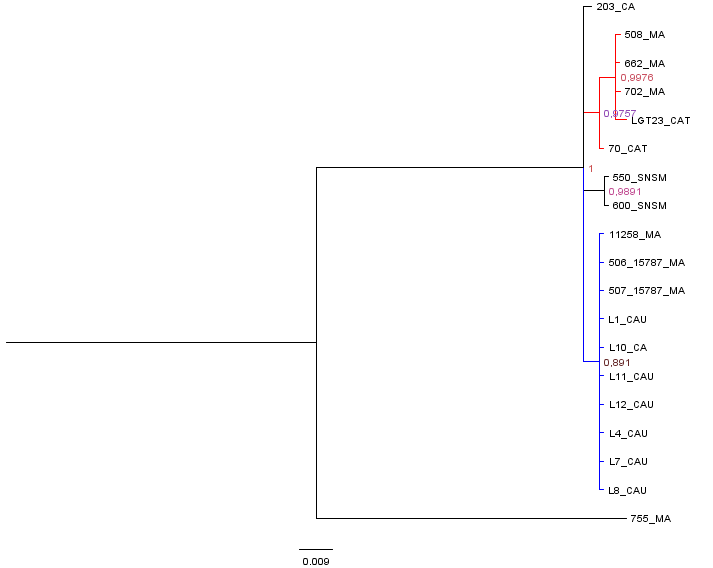


Figure 1. COX1 tree generate in Mr. Bayes, colors are showing the two clades with more than 95% bootstrap. 500000 MCMC generations were ran.


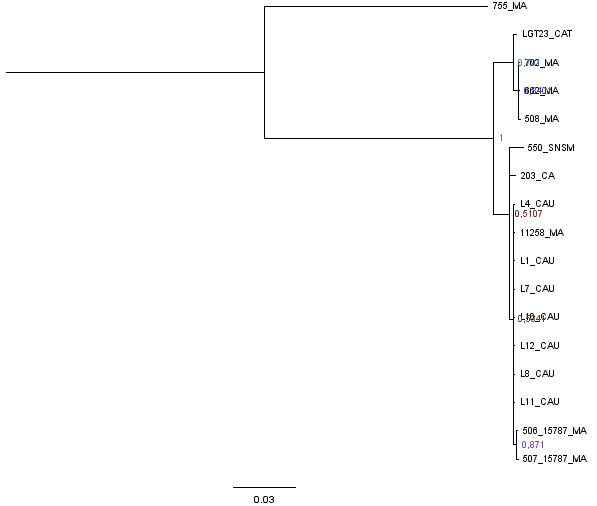


Figure 2. Cytb tree generate in Mr. Bayes, colors are showing the two clades with more than 95% bootstrap. 500000 MCMC generations were ran.


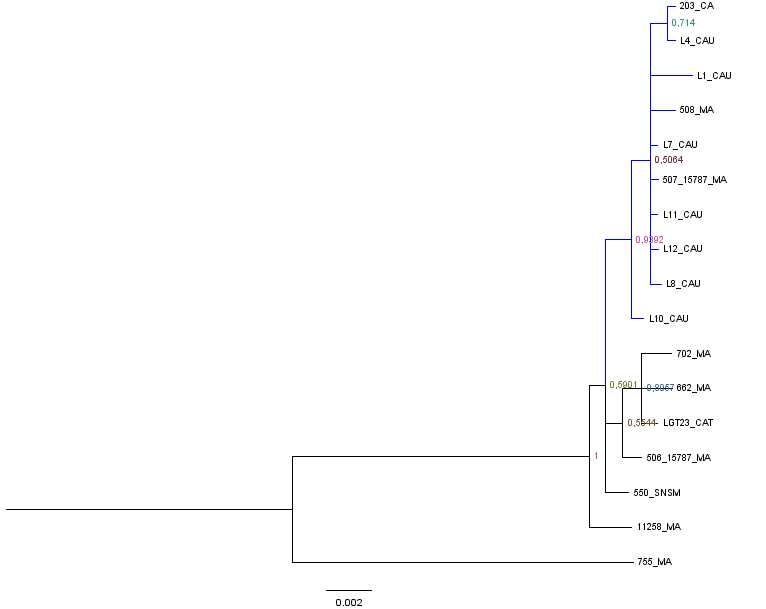


Figure 3. Rag1 tree generate in Mr. Bayes, colors are showing the two clades with more than 95% bootstrap. 500000 MCMC generations were ran.
